# Supplementary material for: YC-1 Antagonizes Wnt/β-Catenin Signaling Through the EBP1 p42 Isoform in Hepatocellular Carcinoma
Source: Cancers (Basel). 2019 May 13;11(5):661. doi: 10.3390/cancers11050661 (PMC6562864; doi:10.3390/cancers11050661)
Supplement: Supplementary file 1 [file cancers-11-00661-s001.pdf]

Supplementary Materials: YC-1 Antagonizes Wnt/β-Catenin Signaling Through the EBP1 p42 Isoform in Hepatocellular Carcinoma

Ju-Yun Wu, Yu-Lueng Shih, Shih-Ping Lin, Tsai-Yuan Hsieh and Ya-Wen Lin

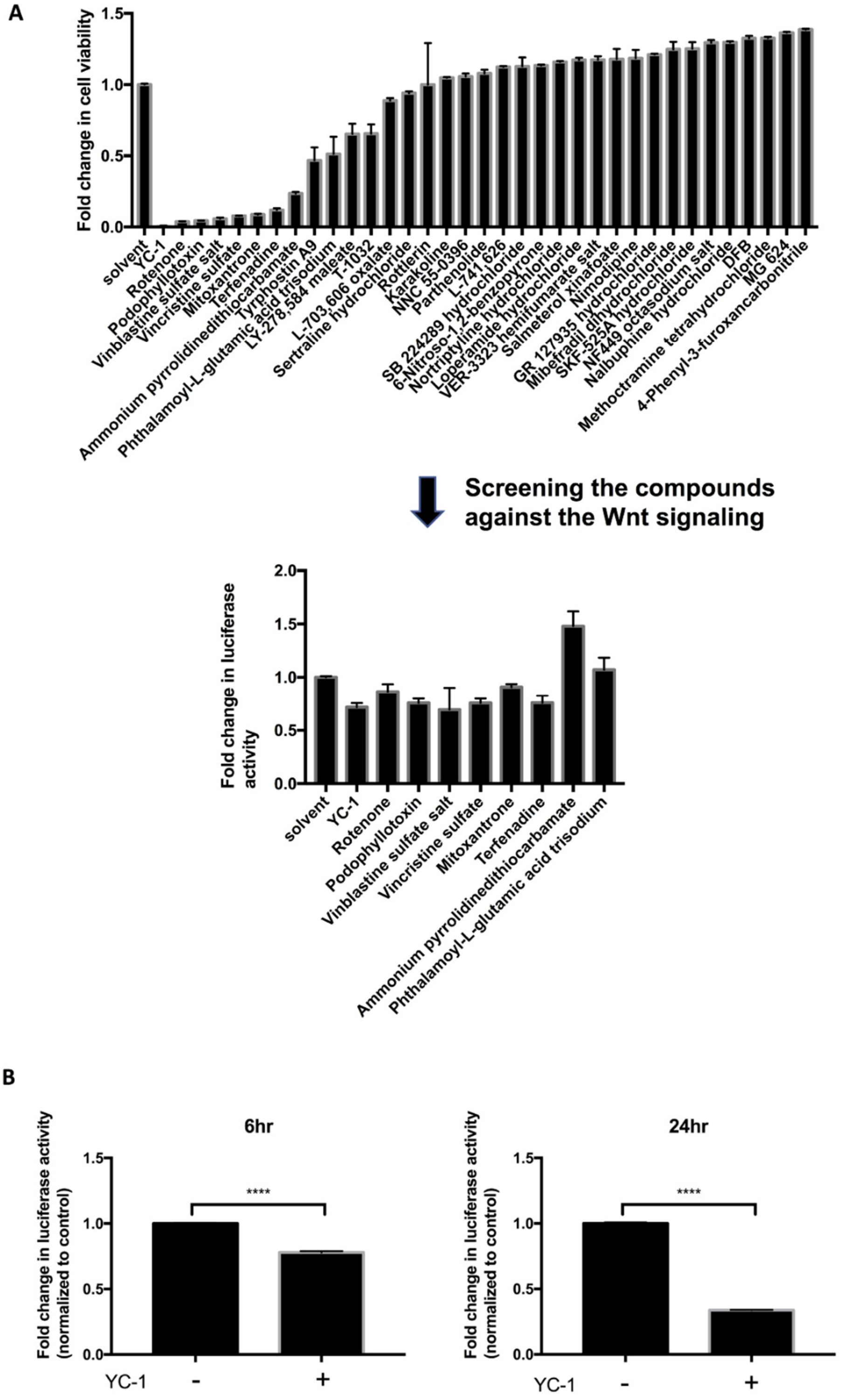

Figure S1. A Wnt-responsive STF luciferase reporter assay was used to identify compounds that inhibited Wnt signaling. The cytotoxic activities of these compounds from the LOPAC library were initially screened. We treated Huh6-M50 cells with each of the compounds from the LOPAC1280 platform at a concentration of 3 μM for 72 hours and then measured cell viability by MTS assays. Subsequently, drugs antagonizing Wnt signaling were identified among the compounds inhibiting the viability of Huh6/M50 cells. We treated Huh6/M50 cells with these cytotoxic compounds at a concentration of 3 μM for 6 hours and measured luciferase activity (**A**). Huh6/M50 cells expressing high levels of luciferase activity were established as the cell model for drug screening. Huh6/M50 cells were treated with YC-1 at a concentration of 3 μM for 6 and 24 hours, and the inhibitory rate of luciferase activity was then analyzed (**B**).

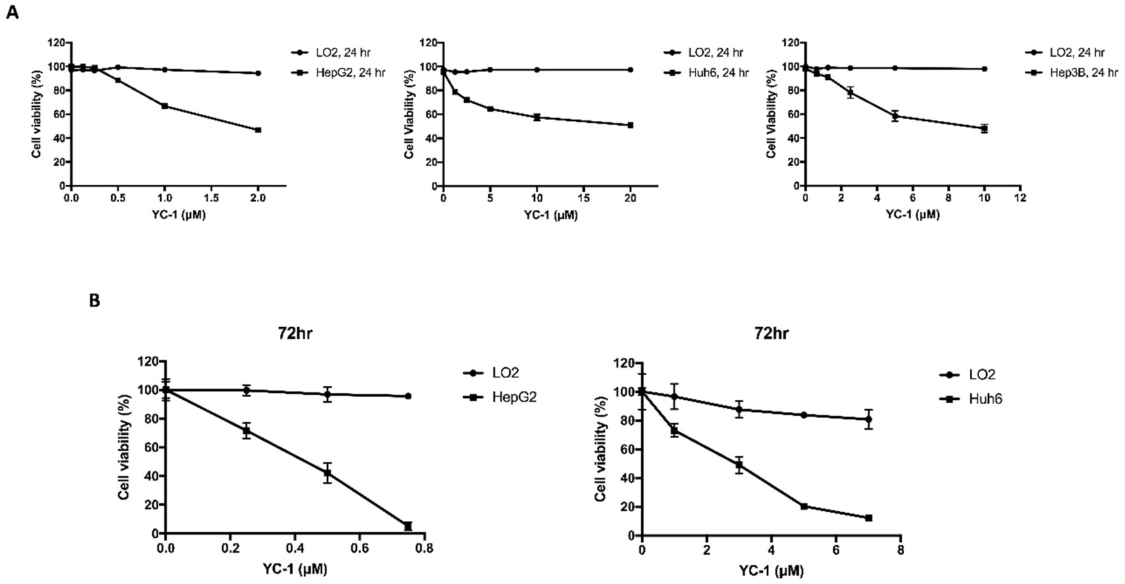

Figure S2. The IC_{50} of YC-1 was determined at 24-hour time point, and YC-1 suppressed HCC cell proliferation for an extended duration. After different concentration of YC-1 treatment, cell viability was measured by MTS assays (**A**). We confirmed the viability of L-02, HepG2 and Huh6 cells after prolonged YC-1 treatment. We treated HepG2 cells with YC-1 at concentrations of 0, 0.25, 0.5 and 0.75 μM for 72 hours. The treatment period for 0, 1, 3, 5, and 7 μM YC-1 in Huh6 cells was extended to 72 hours. Cell viability was measured by MTS assays (**B**).

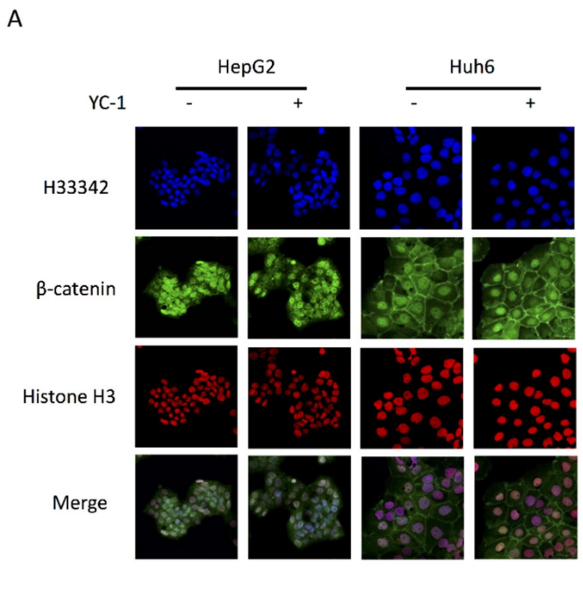

Figure S3. The distribution of β-catenin in HCC cell lines was not appreciably affected upon YC-1 treatment. HepG2 and Huh6 cells were treated with the IC_{50} of YC-1. After 6 hours, cells were fixed with 4% formaldehyde and analyzed by ICC staining and laser scanning confocal microscopy. Hoechst 33342 and an anti-histone H3 antibody were used for nuclear counterstaining (**A**). The levels of β-catenin in the nucleus and cytoplasm were measured by nuclear protein extraction and western blotting (**B**).

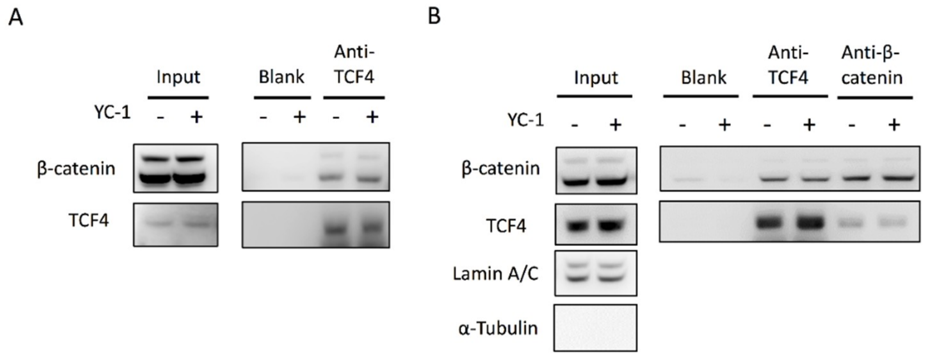

Figure S4. YC-1 treatment did not affect the interaction between β-catenin and TCF4 in HCC cells. HepG2 cells were treated with the IC_{50} of YC-1 for 6 hours. The total lysate from HepG2 cells was isolated and analyzed by co-IP using antibodies specific for TCF4 (**A**). Nuclear protein was extracted from HepG2 cells and analyzed by co-IP using antibodies specific for β-catenin and TCF4 (**B**). Western blotting was used to analyze the immunoprecipitated proteins.

A

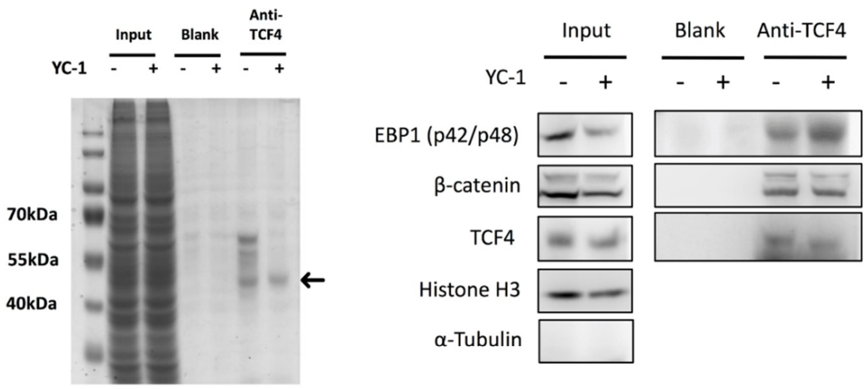

B

Description	Coverage %	Unique Peptides	MW [kDa]
Proliferation-associated protein 2G4 (PA2G4)	11.93	5	43.8
Eukaryotic initiation factor 4A-I (eIF4A)	13.30	3	46.1
Adenosylhomocysteinase (AHCY)	9.26	3	47.7
Rab GDP dissociation inhibitor beta (GDIβ)	6.97	2	50.6
Elongation factor 1-alpha 1 (EEF1A1)	6.06	2	50.1

C

The amino acid sequence of EBP1

1 (N-terminus)
54
MSGEDEQQEQTIAEDLVVTKYKMGGDIANRVLRSLVEASSSGVSVLSLCEKGDA
55
MIMEETGKIFKKEKEMKKGIAFPTSISVNNCVCHFSPLK**SDQDYIL**KEGDLVKIDLG
VHVDGFIANVAHTFVVDVAQGTQVTGRKADVIKAAHLCAEAALRLVKPGNQNT
QVTEAWNKVAHSFNCTPIEGMLSHQLKQHVIDGEK**TIIQNPTDQQK**KDHEKAEF
EVHEVYAVDVLVSSGEGKAKDAGQRTTIYKRDPSKQYGLKMKTSR**AFFSEVER**RF
DAMPFTLRAFEDEKKARMGVVECAKHELLQPFNVLYEK**EGEFVAQFK**FTVLLMP
NGPMRITSGPFEPDLYK**SEMEVQDAELK**ALLQSSASRKTQKKKKKKASKTAENAT
SGETLEENEAGD
394 (C-terminus)

	Unique Peptides	Position
1	SDQDYILK	94-101
2	TIIQNPTDQQK	200-210
3	AFFSEVER	264-271
4	EGEFVAQFK	312-320
5	SEMEVQDAELK	345-355

Figure S5. An antibody-mediated pulldown approach identified candidate β-catenin- or TCF4-interacting proteins, including proliferation-associated protein 2G4 (PA2G4), after YC-1 treatment. HepG2 cells were treated with the IC_{50} of YC-1 for 6 hours, and co-IP experiments were then performed. Anti-TCF4-immunoprecipitated proteins from the cell lysate were detected by Coomassie blue staining. The protein band in the YC-1 group identified by LC-MS/MS is indicated by the arrows (**A**). The screening condition of the identified proteins was dependent on the coverage, number of unique peptides, and molecular weight (**B**). The aligned sequences of the unique peptides were consistent with the amino acid sequence of the protein PA2G4, which is also known as EBP1. The unique peptides were located in the C-terminus and middle regions of EBP1 (**C**).

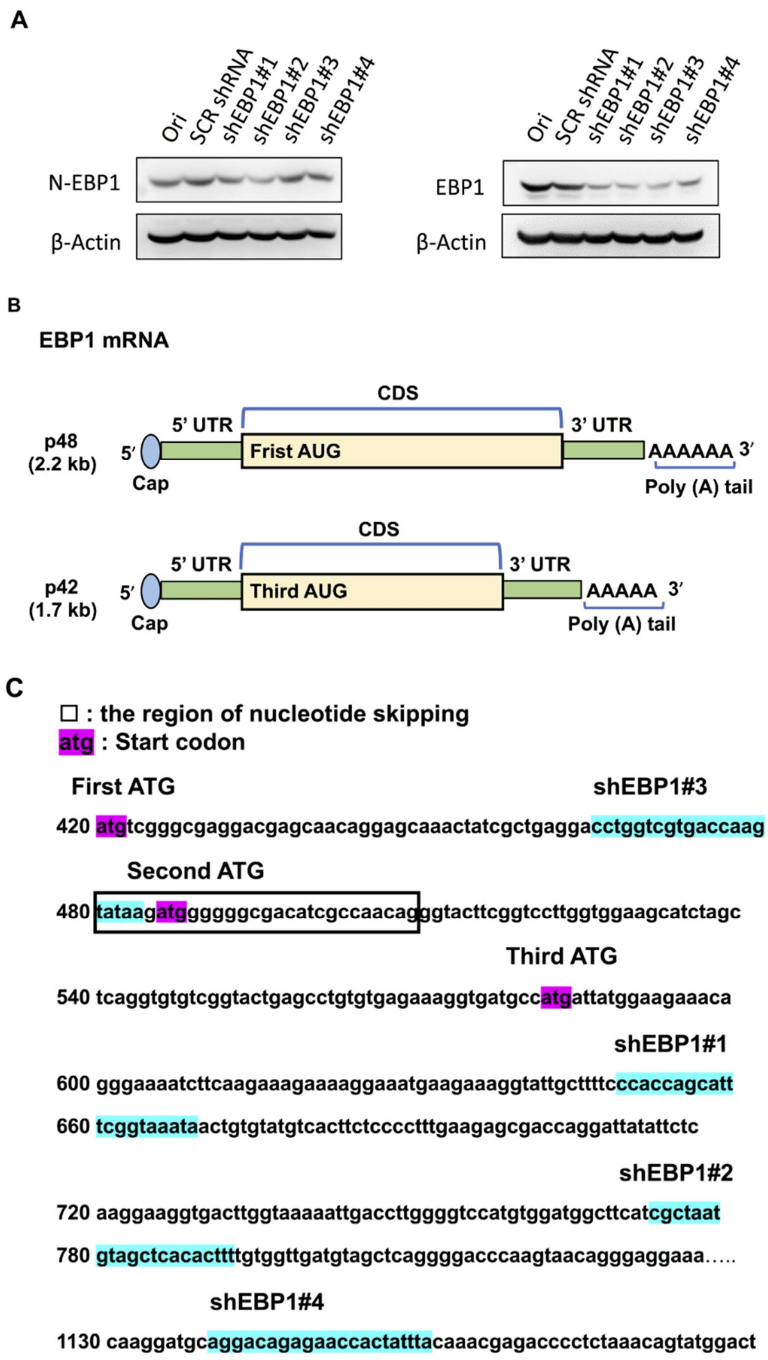

Figure S6. The individual oligo sequences and silencing efficiencies of the EBP1 shRNAs. HepG2 cells were transfected with scrambled shRNA and four different EBP1-specific shRNAs for 24 hours. Protein was extracted from the transfected cells and analyzed by western blotting (**A**). A schematic showing the mRNA transcripts of the EBP1 isoforms. EBP1 pre-mRNA was spliced into two mRNA transcripts because of the skipping of 29 nucleotides. The size of the longer mRNA transcript—the p48 isoform—is 2.2 kb; another transcript is the p42 isoform, with a size of 1.7 kb. The translation of the p48 isoform was initiated at the first AUG codon. The omission of the second AUG codon resulted in a frameshift; thus, translation of the p42 isoform began at the third AUG codon (**B**). The positions targeted by the EBP1 shRNAs are shown in figure (**C**). shEBP1#3 was designed to target the region upstream of the second ATG codon, overlapping the nucleotide skipping region. The other shRNAs were designed to target the region downstream of the second ATG codon (**C**).

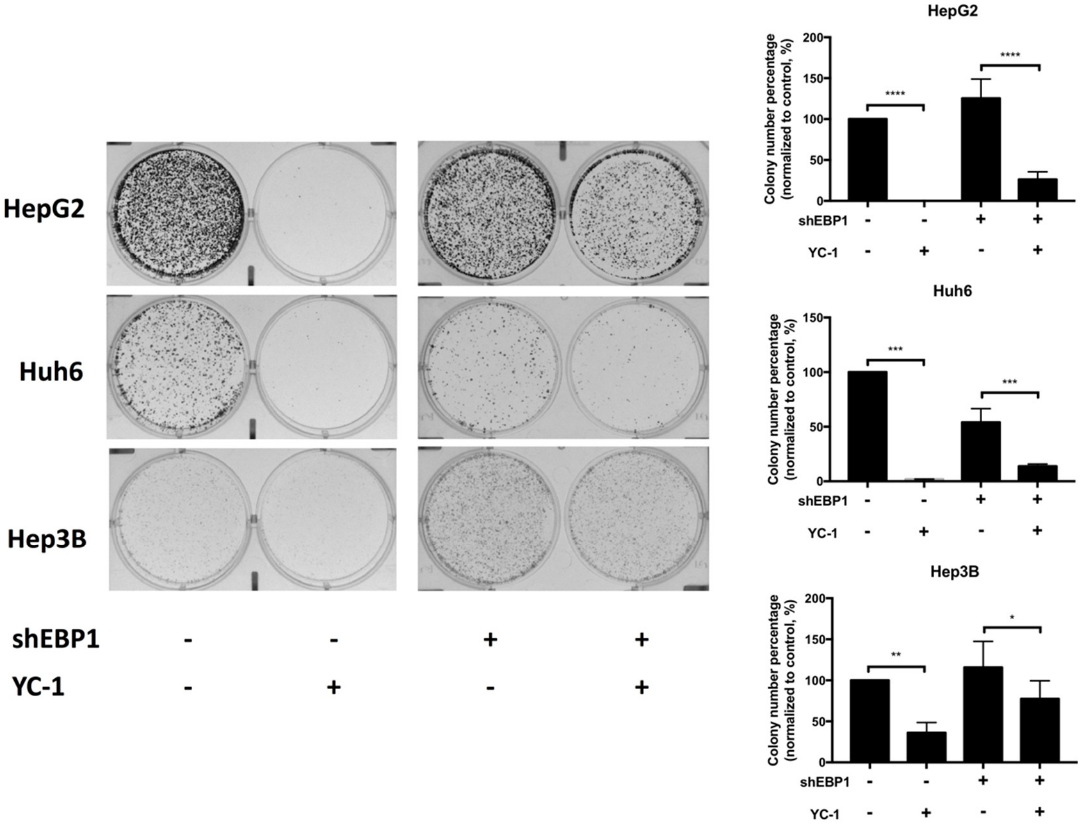

Figure S7. Knockdown of EBP1 by the second EBP1 shRNA inhibited the suppressive effect of YC-1. HCC cells were transfected with scrambled and EBP1-specific shRNA#1 for 24 hours and allowed to recover for 48 hours. The indicated cells were seeded in 6-well plates at 5 × 10^4 cells per well and incubated for 48 hours. After the HCC cells were treated with the IC_{50} of YC-1 for 24 hours, the medium was replaced. The colonies formed by the indicated cells were observed and counted.

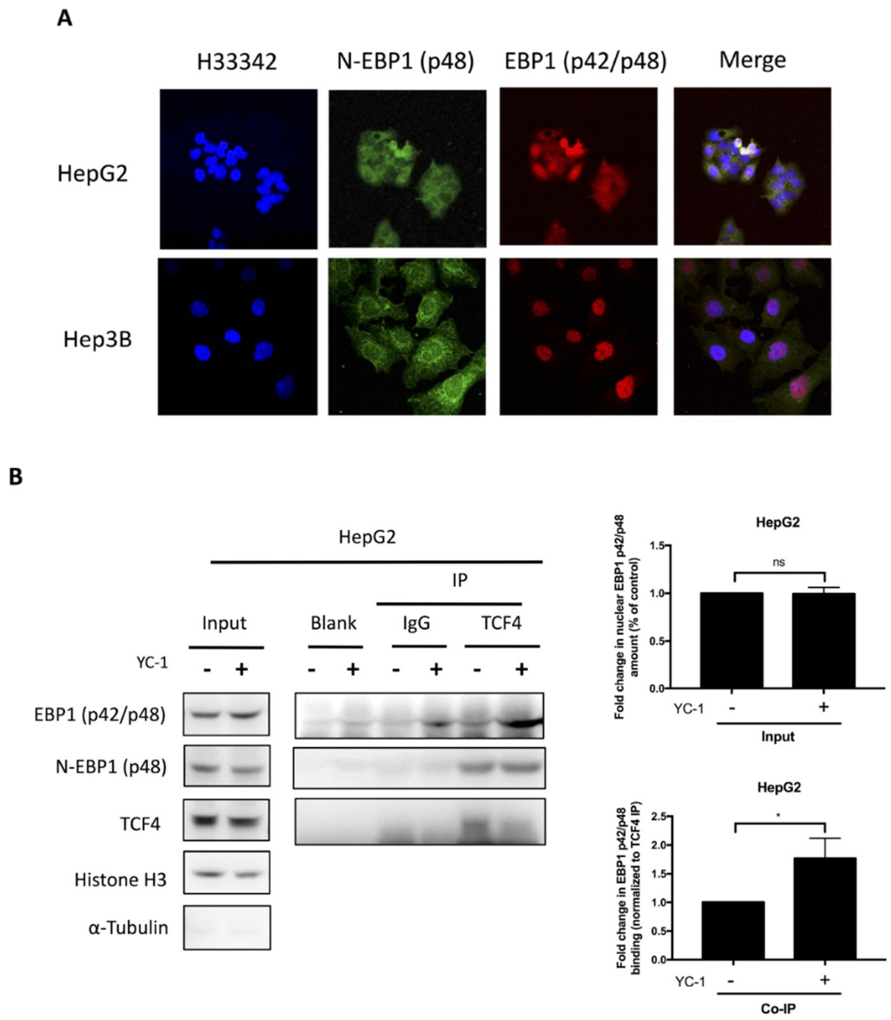

Figure S8. YC-1 treatment did not affect the levels of nuclear EBP1 isoforms but enhanced the interaction of EBP1 with TCF4 in HCC cells. We used ICC staining to detect p42 and p48 and found that both forms were detectable in the cytoplasm and nucleus (**A**). HepG2 cells were treated with YC-1 for 6 hours, and nuclear protein was extracted. The expression of nuclear EBP1 isoforms was measured by western blotting. Nuclear proteins were precipitated by co-IP with anti-TCF4 antibodies, and their expression was then measured by western blotting (**B**).

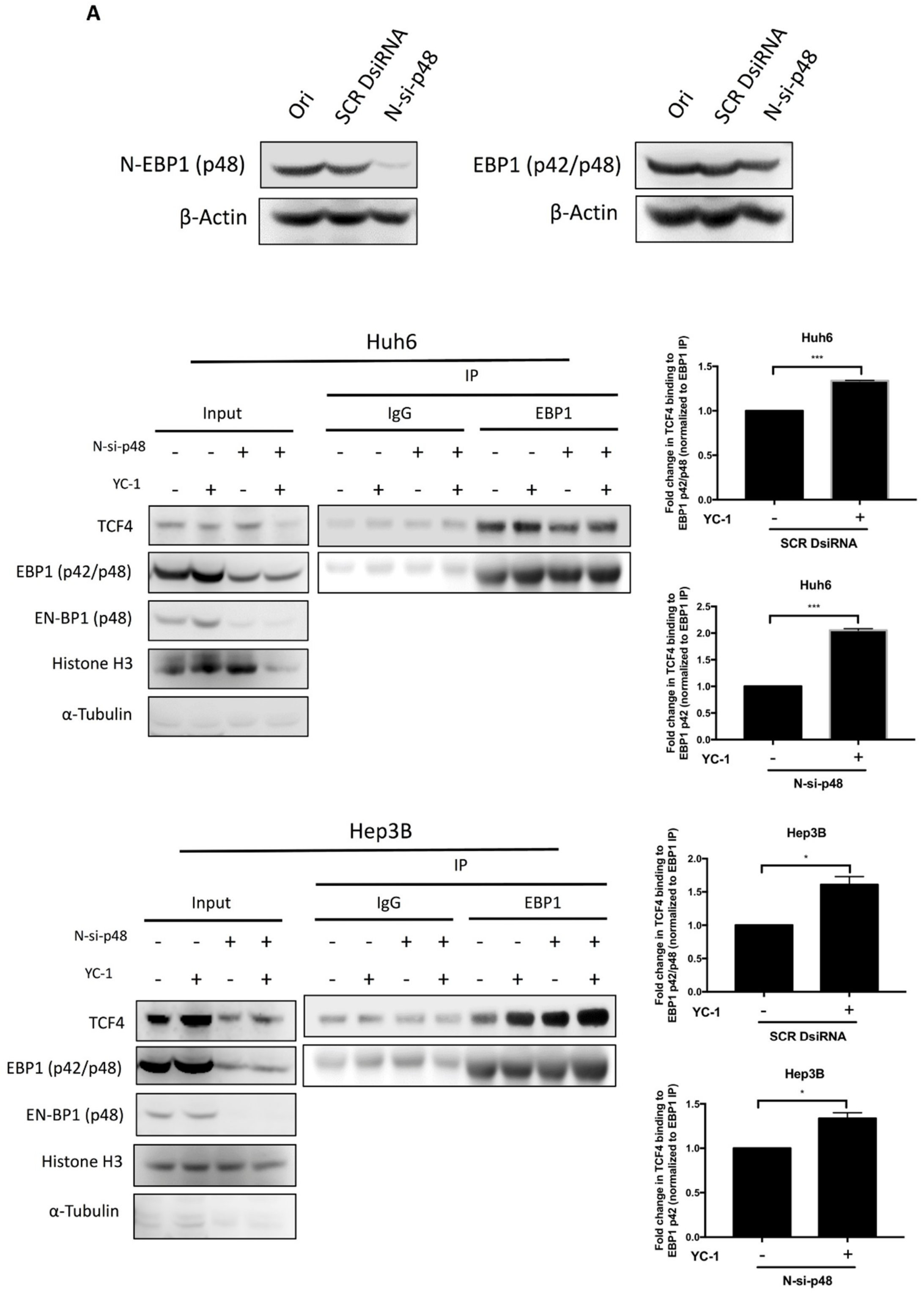

Figure S9. YC-1 enhanced the interaction of EBP1 p42 with TCF4 even in the absence of EBP1 p48. The DsiRNA sequence of N-si-p48 targeting only p48 was designed to recognize the N-terminus of EBP1. HepG2 cells were transfected with scrambled and N-si-p48 constructs. After 48 hours, the expression of N-EBP1 (p48) and EBP1 (p42/p48) was measured by western blotting. After N-si-p48-mediated knockdown, the remaining signal detected by the anti-EBP1 antibody was recognized as primarily EBP1 p42 (**A**). Nuclear proteins from the indicated cells were analyzed with co-IP using anti-EBP1 antibodies. In both the scrambled and N-si-p48 DsiRNA-transfected HCC cells with or

without YC-1 treatment, TCF4 was immunoprecipitated with anti-EBP1 antibodies and detected by western blotting (**B**).

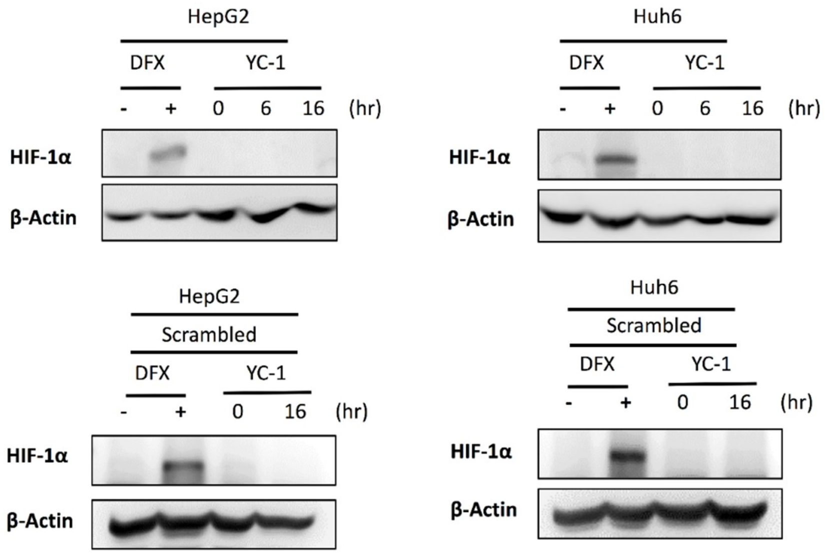

Figure S10. The effect of YC-1 was not associated with HIF-1α. Desferrioxamine (DFX) (Sigma-Aldrich) served as the positive control for HIF-1α induction. Both HepG2 and Huh6 cells were treated with YC-1 following the indicated time course. The YC-1 treatment conditions were consistent between the scrambled shRNA-transfected HepG2 and Huh6 cells. Lysates were collected from cells treated with or without YC-1 and DFX, and the expression of HIF-1α was measured by western blotting.

© 2019 by the authors. Licensee MDPI, Basel, Switzerland. This article is an open access article distributed under the terms and conditions of the Creative Commons Attribution (CC BY) license (http://creativecommons.org/licenses/by/4.0/).
